# Supplementary material for: To denoise or to cluster, that is not the question: optimizing pipelines for COI metabarcoding and metaphylogeography
Source: BMC Bioinformatics. 2021 Apr 5;22:177. doi: 10.1186/s12859-021-04115-6 (PMC8020537; doi:10.1186/s12859-021-04115-6)
Supplement: Supplementary file 1 — Additional file 1 Format: .pdf. Details of the dataset used in the analysis of the article, including information of the sampling localities in the Iberian Peninsula and the sample processing steps prior to sequencing. [file 12859_2021_4115_MOESM1_ESM.pdf]

To denoise or to cluster, that is not the question. Optimizing pipelines for COI metabarcoding and metaphylogeography

A. Antich, C. Palacin, O.S. Wangensteen, X. Turon

Additional file 1

The dataset

We used as a case study an unpublished dataset of COI sequences obtained from benthic communities in 12 locations of the Iberian Mediterranean. These locations are shown in Figure 1. The seaweed-dominated shallow community inhabiting vertical rocky surfaces between -4 and -8 m was sampled by completely scraping off with hammer and chisel standardized surfaces of 25*25 cm. Three replicate samples were taken per location, and all samplings were performed in autumn of 2017.

Sample processing was based on [1] and included a size fractionation step. Extraction and amplification were also performed as in that work using a modified version of the Leray et al [2] primer set (called Leray-XT in [1]), adding also unique 8-bp sample tags at both ends. HTS library preparation was performed using the NextFlex PCR-free DNA-Seq kit (Perkin-Elmer), based on ligation of the Illumina adapters at both ends of the amplicons. See the ms for the implications of PCR-free library construction methods in the application of one of the denoising algorithms (DADA2). We used a full run of a V3 Illumina MiSeq kit with 2*250 bp paired-end sequencing.

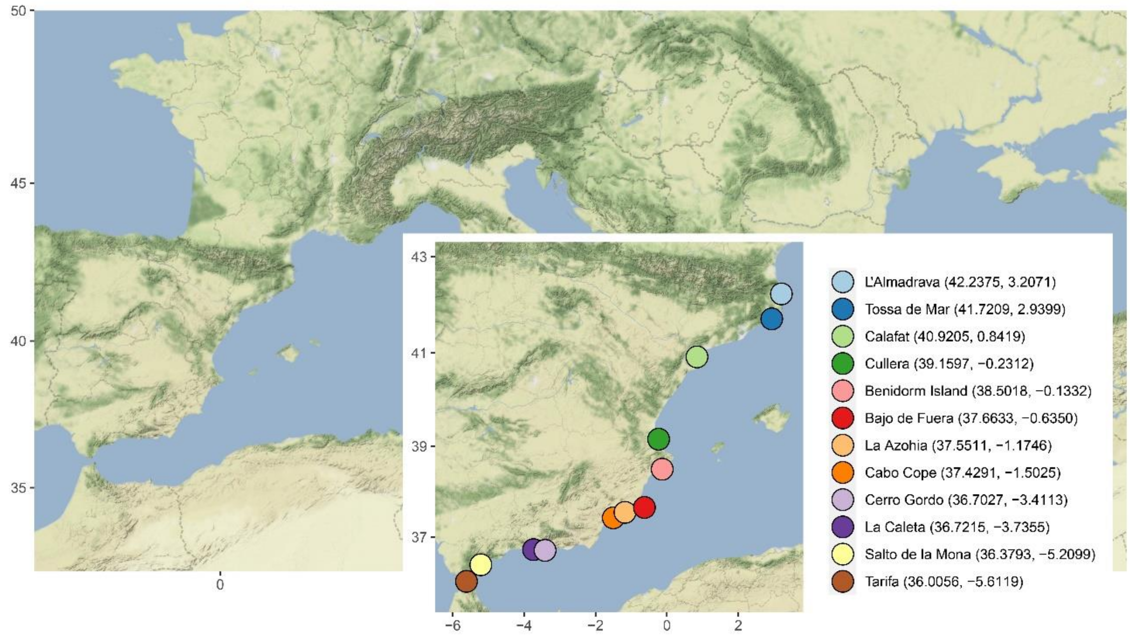

Figure 1. Map of the sampling localities in the Iberian Peninsula, with indication of their coordinates. The map was generated with ggplot [3].

Literature cited

1. Wangensteen OS, Palacin C, Guardiola M, Turon X. DNA metabarcoding of littoral hard-bottom communities: high diversity and database gaps revealed by two molecular markers. Peer J. 2013;6:e4705.
2. Leray M, Yang JY, Meyer CP, Mills SC, Agudelo N, Ranwez V, Boehm JT, Machida RJ. A new versatile primer set targeting a short fragment of the mitochondrial COI region for metabarcoding metazoan diversity: application for characterizing coral reef fish gut contents. Frontiers in Zoology. 2013;10:34.
3. Kahle D, Wickham H. ggmap: spatial visualization with ggplot2. The R Journal. 2013;5(1):144-161.
