# Supplementary material for: To denoise or to cluster, that is not the question: optimizing pipelines for COI metabarcoding and metaphylogeography
Source: BMC Bioinformatics. 2021 Apr 5;22:177. doi: 10.1186/s12859-021-04115-6 (PMC8020537; doi:10.1186/s12859-021-04115-6)
Supplement: Supplementary file 3 — Additional file 3 Format: .pdf. Details of the taxonomic benchmarking. [file 12859_2021_4115_MOESM3_ESM.pdf]

To denoise or to cluster, that is not the question. Optimizing pipelines for COI metabarcoding and metaphylogeography

A. Antich, C. Palacin, O.S. Wangensteen, X. Turon

Additional file 3

Taxonomic benchmarking

We combined all unique ESVs retained after the denoising algorithms (those retained by the different versions of DnoisE and those retained by DADA2, for a total of 116,218 ESVs) and assigned them taxonomically with ecotag. We found that 25,197 sequences had a species-level assignment, comprising 690 species, of which 187 were represented by a single sequence. We further refined this dataset by accepting only sequences whose best hit in the reference database was ≥ 0.97, which is in accordance with the mean intra-MOTU distance we found in our dataset with SWARM. This pruned dataset (henceforth species-level dataset, available as Additional file 4), consisted of 14,487 assigned sequences belonging to 422 species, with 130 having only one sequence. Without the inclusion of the entropy-corrected ESV dataset (which kept more ESVs than the other methods) we had 5,147 sequences assigned at species level, belonging to 417 species. Thus, the inclusion of this dataset almost tripled the number of sequences with species-level hit but these represented only five extra species with respect to the other datasets, indicating that the gain in ESVs in the entropy-corrected procedure mainly increases within-MOTU variability.

We checked how many of the sequences in the species-level dataset were recovered with the different denoising and clustering methods. We also assessed whether these sequences were grouped in closed MOTUs (meaning all sequences in the MOTU belonged to the same species and no other sequences of this species were found in other MOTUs), open MOTUs (i.e., all sequences belonged to the same species, but not all sequences assigned to the species were included) and hybrid MOTUs. The latter included MOTUs with sequences assigned to more than one species, or MOTUs with a combination of sequences assigned to one species and sequences not in the species-list dataset (i.e., they don't have species-level assignment, or they do with less than 97% similarity). Closed MOTUs were further subdivided among those with only one sequence (closed singleton) and those with several sequences (closed group).

The proportion of the 422 species that were recovered by the different methods was in all cases high (above 96%), but the datasets denoised with DADA2 featured the lowest proportions (Figure 1). The entropy-corrected datasets, on the other hand, recovered all species. Differences were also apparent in the proportion of ESVs with species-level assignment that were found in the different datasets. In general, DADA2-based datasets had a lower proportion of ESVs with species assignment indicating that more sequences assignable to species have been merged during denoising. Clustering first reduced appreciably this proportion in the UNOISE3-based datasets (Du_S vs. S_Du, ca. 13% reduction), while it didn't vary in the comparison Da_S vs S_Da. Entropy-corrected datasets had not only a higher number of ESVs, but a higher proportion of them (>11%) with species-level assignment (Figure 1).

As shown in Figure 2, when we checked the different datasets generated, irrespective of the method the majority (62-75%) of MOTUs that had sequences assigned to the species rank were closed, 7-10% were open, and 18-28% were hybrid MOTUs. This indicates that, in all cases, the denoising plus clustering methods performed reasonably well in recovering species that were identified as such in taxonomic assignment of the ESVs. The UNOISE3 algorithm, however, recovered ca. 60% more closed group MOTUs that DADA2, and the opposite occurred for closed singleton MOTUs, for a similar total. This is the result of the higher number of sequences retained by UNOISE3, that translated into a higher ability to recover MOTUs with internal diversity. The proportion of hybrid MOTUs was lower in the DADA2 denoised datasets, which were the most stringent in terms of ESVs retained, while the datasets with entropy correction, the ones with higher number of ESVs, featured a slightly higher proportion of hybrid MOTUs than those not-corrected. We verified manually these hybrid MOTUs, and in most cases they were due to the inclusion of some sequences not in the species-level dataset (for instance, sequences with less than 97% similarity with their matches in the reference database), rather than to the lumping of sequences assigned to different species. To check this point, we repeated the analysis without enforcing the 97% similarity, and the proportion of hybrid MOTUs decreased by half. This indicates that in many cases the hybrid MOTUs found in the 97% restricted-similarity analysis comprised sequences assigned by ecotag to the same species, but some of them with similarity levels lower than 97%. Overall, then, the taxonomic benchmarking showed a good correlation between MOTUs and species assignments performed with ecotag.

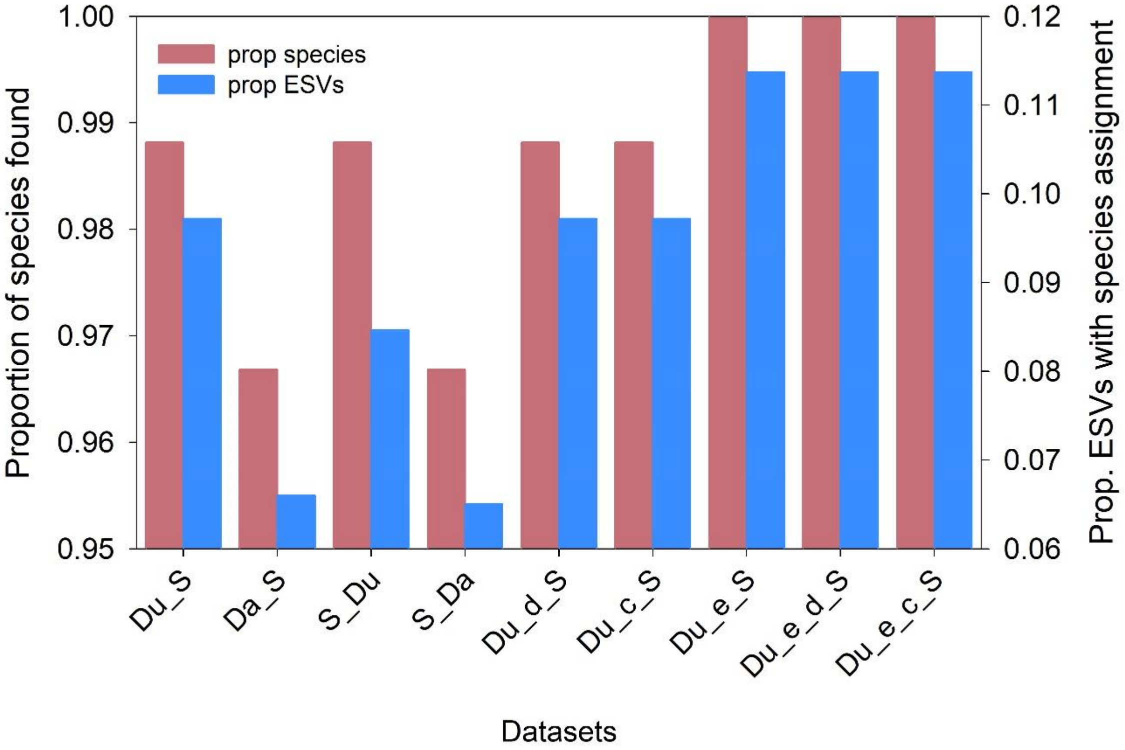

Figure 1. Proportion of the species in the species-level dataset recovered and proportion of ESVs with species-level assignment found in the different datasets.

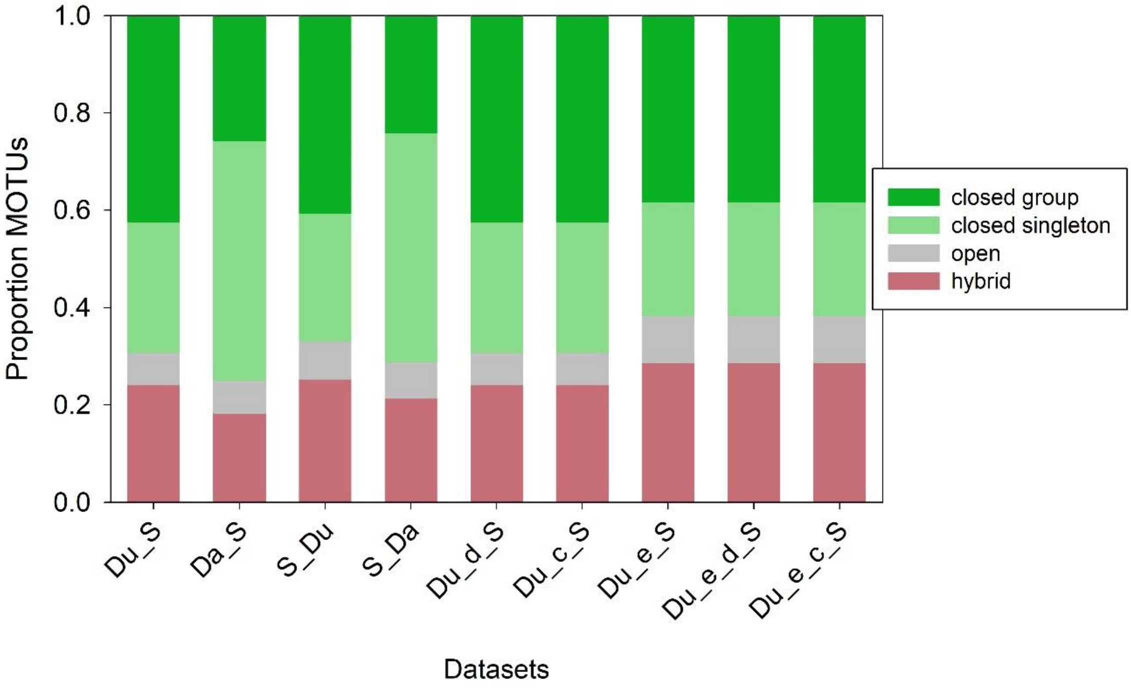

Figure 2. Proportion of closed, open, and hybrid MOTUs found in the different datasets
